# The Structure of Sucrose-Soaked Levansucrase Crystals from *Erwinia tasmaniensis* reveals a Binding Pocket for Levanbiose

**DOI:** 10.3390/ijms21010083

**Published:** 2019-12-20

**Authors:** Ivan Polsinelli, Rosanna Caliandro, Nicola Demitri, Stefano Benini

**Affiliations:** 1 Bioorganic Chemistry and Bio-Crystallography laboratory (B2Cl), Faculty of Science and Technology, Free University of Bolzano, Piazza Università 5, 39100 Bolzano, Italy; ivan.polsinelli@unibz.it (I.P.); rosanna.caliandro@unibz.it (R.C.); 2 Elettra-Sincrotrone Trieste, S.S. 14 Km 163.5 in Area Science Park, Basovizza, 34149 Trieste, Italy; nicola.demitri@elettra.eu

**Keywords:** glycoside hydrolase, GH68, fructosyltransferase, fructooligosaccharides, FOS biosynthesis, prebiotic oligosaccharides

## Abstract

Given its potential role in the synthesis of novel prebiotics and applications in the pharmaceutical industry, a strong interest has developed in the enzyme levansucrase (LSC, EC 2.4.1.10). LSC catalyzes both the hydrolysis of sucrose (or sucroselike substrates) and the transfructosylation of a wide range of acceptors. LSC from the Gram-negative bacterium *Erwinia tasmaniensis* (EtLSC) is an interesting biocatalyst due to its high-yield production of fructooligosaccharides (FOSs). In order to learn more about the process of chain elongation, we obtained the crystal structure of EtLSC in complex with levanbiose (LBS). LBS is an FOS intermediate formed during the synthesis of longer-chain FOSs and levan. Analysis of the LBS binding pocket revealed that its structure was conserved in several related species. The binding pocket discovered in this crystal structure is an ideal target for future mutagenesis studies in order to understand its biological relevance and to engineer LSCs into tailored products.

## 1. Introduction

In the last decade, interest has grown towards levan/inulin oligosaccharides. They have a wide range of applications, from personal care to packaging. These molecules are especially relevant for their medical applications and prebiotic activity [1,2,3,4,5].

Levansucrases (LSCs, EC: 2.4.1.10) and inulosucrases (INUs, EC: 2.4.1.9) are major fructosyltransferases employed as biocatalysts in the synthesis of fructans and fructooligosaccharides (FOSs). Both are members of glycosyl hydrolase family 68 (GH68) [6]. LSC catalyzes the transfructosylation of the fructose component of sucrose by using a variety of acceptor molecules, forming β-(2,6)-linked oligofructans. When a water molecule acts as an acceptor, the reaction results in the hydrolysis of sucrose into glucose and fructose [1].

LSCs are used in the fermentative production of microbial oligosaccharides and polysaccharides due to their ability to interact with low-cost substitutes of sucrose, e.g., syrups and molasses [7,8].

The existence of a wide spectrum of nonconventional fructosyl acceptors explains the biotechnological interest in LSCs. These enzymes can interact with nonconventional fructosyl acceptors and donors [9], such as monosaccharides, disaccharides, and sucrose homologs. For example, LSCs from *Pseudomonas syringae* pv. tomato DC3000 and *Pseudomonas aurantiaca* are able to transfructosylate deoxy sugars or alditols such as fucose, ribose, sorbitol, and xylitol [10]. Among nonconventional substrates, lactose has been one of the most extensively studied. This is due to its combined role with sucrose in a reaction catalyzed by LSCs from *Bacillus spp.* (*B. methylotrophicus* SK21.002 [11], *B. subtilis* NCIMB 11871 [12], and *B. licheniformis* [13]) to produce lactosucrose, which is a trisaccharide with prebiotic activity [14]. 

Aromatic alcohols such as phenol derivatives (e.g., hydroquinone) [15] and isoflavones (e.g., puerarin) [16] can also be transfructosylated by LSCs from *B. subtilis* (SacB, BsLSC) and *Gluconacetobacter diazotrophicus* (GdLSC), respectively. The improved physical, chemical, and bioactive properties (solubility, stability, availability, and activity) of these glycosides make them relevant to pharmaceutical applications.

In the last decade, several studies have been carried out to understand which residues are the most relevant in the reaction mechanism [2]. Thanks to these studies, engineered glycosyltransferases can be used to obtain specific compounds such as FOSs (e.g., 6-nystose) instead of high-molecular weight (HMW) levan [17].

To better describe the relevant residues, the active site of LSC is commonly divided into layers. Moving from the sucrose binding site outwards, there are three layers: the first, second, and third. Mutations S173A, S173G, and S422A (first layer) in the LSC of *Bacillus megaterium* (BmLSC, PDB ID: 3OM7) increase transfructosylate activity by 194%, 53%, and 42%, respectively [18]. In *P. syringae* pv. tomato, LSC3 with the E146Q mutation (second layer) exhibits increased production of FOSs compared to wildtype [19]. SacB of *B. subtilis* with a Y429N mutation (second layer) has mostly hydrolytic activity and can produce short-chain FOSs instead of HMW levan [20].

Due to the high-yield production of FOSs, the product spectrum and well-optimized production of recombinant enzyme in *Escherichia coli*, LSCs from *Erwinia tasmaniensis* (EtLSC) [21] and *Erwinia amylovora* (EaLSC) [22,23] are interesting candidates for engineered LSCs that produce tailor-made fructans.

The structure of LSC is known in *B. subtilis*, *B. megaterium*, *E. amylovora*, *E. tasmaniensis*, and *G. diazotrophicus*. LSCs have similar structures, and their active sites possess common structural features [24], such as the triad of amino acids involved in catalysis (Asp46, Asp203, and Glu287 in EaLSC) [25]. LSC has been successfully crystallized in complex with sucrose (*B. subtilis*, Protein Data Bank (PDB) ID: 1PT2), raffinose (*B. subtilis*, PDB ID: 3BYN), fructose, and glucose (*E. amylovora,* PDB ID: 4D47). While the sucrose binding site is conserved, superficial areas and volumes vary across species due to variability in the surrounding loops [2,25].

In this report, we present the first known crystal structure of an LSC, EtLSC, in complex with levanbiose (LBS). LBS is an intermediate in the synthesis of oligolevans in the LSC enzyme. The complex was obtained by soaking EtLSC apo crystals (PDB ID: 6FRW) in a concentrated solution of sucrose (0.5 M) in order to trap reaction intermediates/products in the crystals. We describe an unexplored plausible binding site for LBS. We analyzed conserved amino acids in the binding pocket of LBS and compared their structural arrangement to other LSCs from Gram-positive and -negative bacteria. The aims of these analyses were to understand the biological relevance of the binding pocket and explore possible implications for LSC engineering.

## 2. Results and Discussion

The structure of LSC from *E. tasmaniensis* in complex with LBS was determined with a maximum resolution of 1.58 Å (space group *P 4_1_2_1_2*). Data collection and structure-refinement statistics are summarized in Table 1. Atomic coordinates and experimental-structure factors were deposited in the PDB with PDB ID: 6RV5.

Overall, the protein structure showed great similarity with apo EtLSC (PDB ID: 6FRW) [21] and its closest homolog EaLSC in complex with glucose and fructose (PDB ID: 4D47) [25]. 

Both EtLSC and EaLSC act via a distributive (nonprocessive) mechanism. This mechanism is known to produce low-molecular weight (LMW) levan and a mixture of FOSs [2]. For example, EaLSC and EtLSC mainly produce short-chain FOSs with 3–6 degrees of polymerization (DP) [21,22], while the main product of GdLSC is 1-kestose [24,26]. Furthermore, BmLSC produces FOSs with a DP ranging from 2 to 20 [27]. Even SacB can catalyze the formation of LMW levan under conditions that favor a nonprocessive mechanism [28].

The complex process of polymerization is the main factor that determines the wide range of products synthesized by LSC. Specifically, the DP is increased via a cycle of fructosyl capture, transfer, and release of fructosylated intermediates. These intermediates belong to one of two main types of FOS, n fructose units with glucose moiety (GF_n_) or exclusively n fructose units (F_n_). LBS, a fructose dimer, is an intermediate that seems to be produced in the late phase of the reaction. It was described as a secondary intermediate in the LSC from *B. subtilis* (SacB) [28].

Both types of FOS (GF_n_ and F_n_) have been found in the product mixture from EtLSC, and the following species were identified: Levanbiose (F_2_), levantriose (F_3_), 6-kestose (GF_2_), 6-nystose (GF_3_), and 6,6,6-kestopentaose (GF_4_) [21].

**Figure 1:**
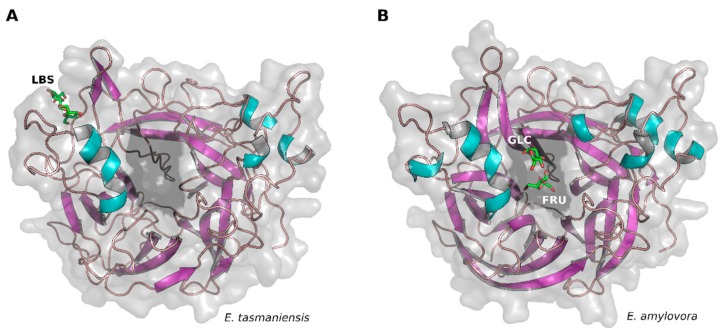
Comparison of ligand location in levansucrases (LSCs) from *Erwinia tasmaniensis* and *Erwinia amylovora*. (**A**) Cartoon representation of *E. tasmaniensis* LSC (EtLSC) structure with levanbiose (LBS) bound (PDB ID: 6RV5). Note: ligand molecule shown as green stick; active-site surface highlighted in black. (**B**) Cartoon representation of *E. amylovora* LSC (EaLSC) structure with fructose and glucose bound (PDB ID: 4D47). Note: ligands (hydrolysis products) shown as green sticks; active-site surface highlighted in black.

A comparison of the structure of EtLSC (this report, PDB ID: 6RV5) and EaLSC (PDB ID: 4D47) was obtained by applying the same soaking procedure with sucrose but with different soaking times, revealing that different products bind to different locations on the enzyme. Although there was similarity between the products of hydrolysis (glucose and fructose) trapped in EaLSC [25] and LBS trapped in EtLSC (PDB ID: 6RV5), these molecules did not bind in similar pockets. In fact, LBS was bound to an exposed pocket on the surface of EtLSC (Figure 1A) while the products of hydrolysis were located inside the active site of EaLSC (Figure 1B). The active site was within the inner part of the β-propeller, which is also found in other LSCs. 

**Figure 2:**
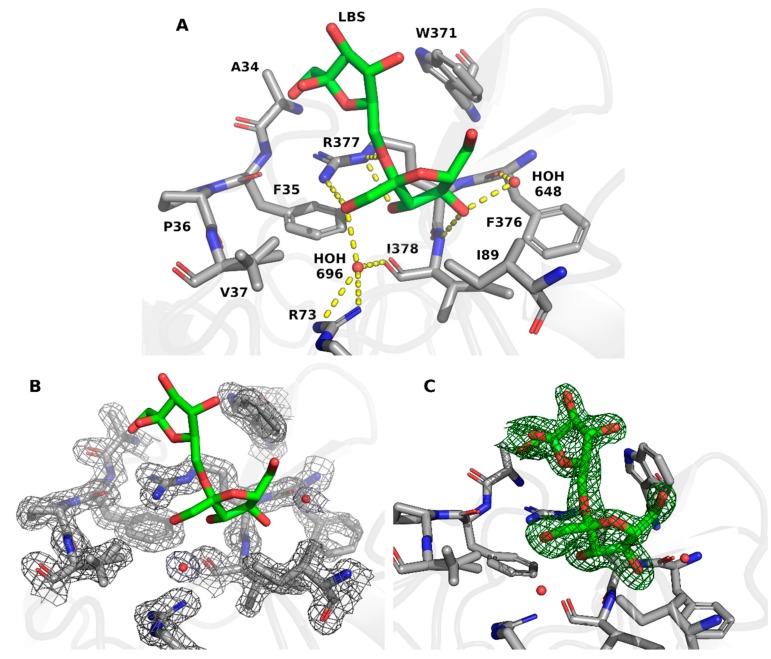
LBS binding pocket. (**A**) LBS interaction with EtLSC (PDB ID: 6RV5). Note: ligand molecule shown as green stick. (**B**) Representation of 2*F*_obs_−*F*_calc_ electron density map of the LBS binding site. Note: electron-density map contoured at 1.5 σ. (**C**) Representation of polder map (omit map that excludes bulk solvent around omitted region) calculated with exclusion of LBS molecule. Note: polder map contoured at 4 σ.

The complex of LBS with EtLSC was formed during the soaking of protein crystals with sucrose. It was located in a small pocket defined by residues Ala34, Phe35, Pro36, Val37, Arg73, Ile89, Trp371, Phe376, Arg377, and Ile378 (Figure 2A). LBS formed three hydrogen bonds with the Arg377 sidechain—LBS O1 interacted with Nη2, while LBS O2 and O3 interacted with Nε. LBS O4 formed another hydrogen bond with the N atom of the Ile378 backbone. Other residues in the pocket interacted with a nonpolar part of the molecule through hydrophobic bonds.

Two water molecules were present in the pocket. The first water molecule (residue 648 in the PDB) formed hydrogen bonds with LBS O4 and the mainchain oxygen of Phe376. Backbone O of Ile378, Arg73 Nη1/Nη2, and LBS O1 formed bonds with a second water molecule (residue 696 in the PDB). After model building (see material and methods section) the residues and the waters of the binding pocket clearly fit the 2*Fobs−Fcalc* electron density map (Figure 2B) and the LBS perfectly fit the unbiased omit Polder map (Figure 2C)

**Figure 3:**
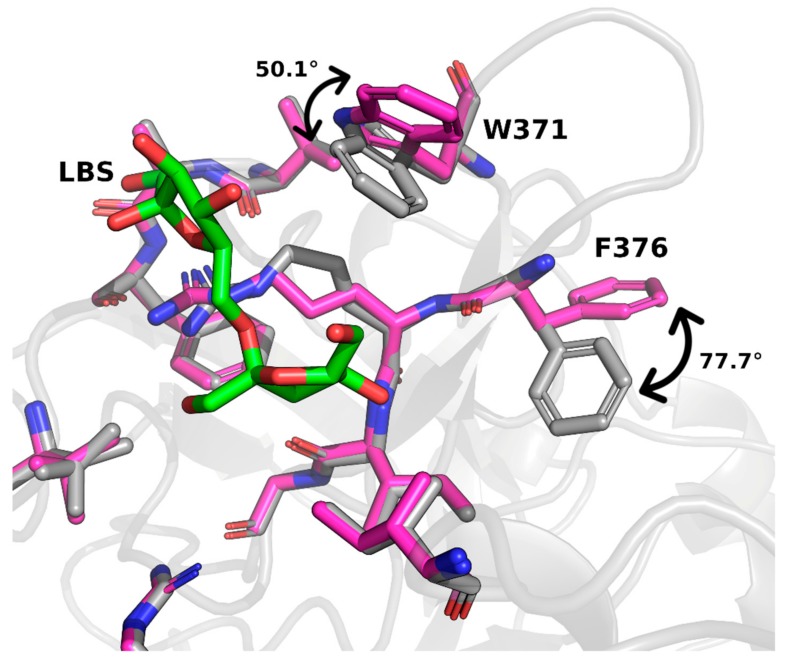
Residue movement upon LBS binding. Relevant conformational changes involved residues W371 and F376. Note: magenta, EtLSC structure with LBS; grey, apo enzyme (PDB ID: 6FRW).

LBS binding caused two noticeable movements in the sidechains of residues located in its binding pocket. This was remarkable when compared with the EtLSC apo structure (PDB ID: 6FRW) sharing the same crystallization conditions. The indole ring of the Trp371 sidechain tilted approximately 50°, and the benzyl moiety of Phe376 flipped 77.7°. Both residues moved toward the LBS moiety in the pocket (Figure 3). The movement of these two residues suggests that binding is mediated by one of the two fructose units in LBS.

Considering all the LSC studies to date, it can be concluded that LSCs from Gram-negative bacteria produce short-chain FOSs, while those from Gram-positive bacteria produce longer-chain oligosaccharides or levan (either LMW or HMW). Some structural features have been proven to be correlated with the length of the product, and are therefore different in Gram-negative and -positive bacteria [25]. For example, an arginine-to-histidine mutation at position 360 in SacB has been proposed as a switch in the production of either long- or short-chain FOSs [29]. Arginine is substituted by histidine in the LSCs of Gram-negative bacteria, including EaLSC and EtLSC, without losing the ability to perform the transfructosylation reaction.

**Figure 4:**
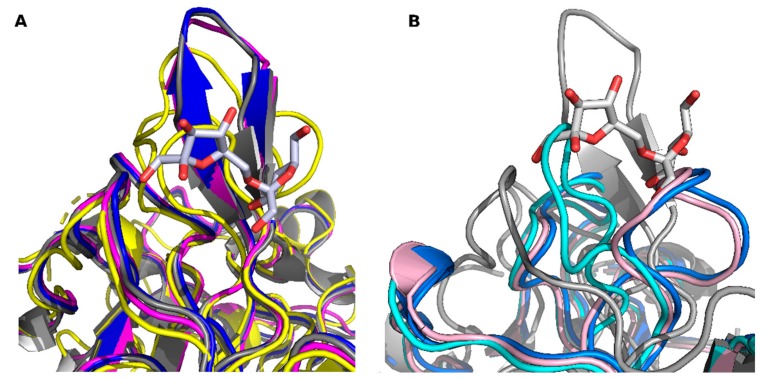
Comparison of LBS binding pocket in different LSC structures. (**A**) Proposed binding of LBS to structures with similar loop conformations. Note: grey and blue, *E. tasmaniensis* (PDB ID: 6RV5 and 6FRW respectively); magenta, *E. amylovora* (PDB ID: 4D47); and yellow, *Gluconacetobacter diazotrophicus* (PDB ID: 1W18). (**B**) Superimposition of LBS on structures with different loop conformations. Note: marine blue, *B. subtilis* (PDB ID: 1OYG); cyan and pink, *B. megaterium* (PDB ID: 3OM7 and 3OM2, respectively).

It has previously been proposed that the loop formed by residues 366–380 contain residues that are able to shape the product spectrum of LSCs [25]. We compared the loop formed by residues 368–378 in EtLSC with other LSC structures available in the PDB (Figure 4A,B). From examining the available crystal structures, it is clear that the presence of LBS in the analyzed region could only be compatible with LSCs from Gram-negative bacteria. The loop conformation in structures of *E. amylovora* [25] and *G. diazotrophicus* [24] could allow for the presence of an LBS molecule (Figure 4A). In contrast, loop conformation is incompatible with LBS binding in the LSCs from Gram-positive bacteria *B. subtilis* [30] and *B. megaterium* [31] due to a ligand clash (Figure 4B). However, loop conformations could be influenced by crystal packing, and their variability could be limited. This may be explained by the involvement of loops in crystal contacts, as in the case of BmLSC. As a solution, loops could adopt slightly different conformations to allow for the required flexibility.

Nevertheless, conformations of loops surrounding the sucrose binding site [25], the presence of an LBS binding site, and other structural features may suggest a correlation with the synthesis of short-chain FOSs in Gram-negative bacteria. The chain length of fructans is determined by enzyme concentration and consequently by enzyme-product interactions [28]. Therefore, a stable LSC–LBS intermediate could favor the production of shorter-chain FOSs. The presence of LBS in the superficial pocket might also favor contacts between adjacent LSC molecules in the solution and increase enzyme density, thereby enhancing the probability of enzyme-product interactions in the active site. The ability to hold small oligofructans (e.g., LBS) in close vicinity to the active site of the enzyme may increase the likelihood that these molecules are used as fructose acceptors that therefore increase the production of small/medium DP oligosaccharides.

However, LSC structures currently available in the PDB belong to a restricted number of organisms, and further studies are required to gain a clear understanding of the differences between Gram-positive and -negative bacteria. Furthermore, the mechanism behind LBS migration towards or away from the active site, and its effect on the spectrum of generated products is still unclear.

## 3. Materials and Methods

The production of recombinant LSC from *E. tasmaniensis* (strain *Et1*/*99*) and its crystallization have been previously described [21]. In brief, the PCR-amplified gene was cloned into a pMCSG49 vector [32] and then expressed in *E. coli* BL21(DE3) star pLysS cells. Purified EtLSC was concentrated to 10 mg/mL (20 mM HEPES pH 7.5, 150 mM NaCl) and used for crystallization.

The crystals were grown by hanging drop vapor diffusion from drops consisting of 1 μL EtLSC solution and 1 μL precipitants (28% glycerol, 14% PEG4000, 2.5 mM manganese(II) chloride tetrahydrate, 2.5 mM cobalt(II) chloride hexahydrate, 2.5 mM nickel(II) chloride hexahydrate, and 2.5 mM zinc acetate dihydrate). The crystals were then soaked in a sucrose-containing solution corresponding to the crystallization drop to a final concentration of 0.5 M at different soaking times. Following this, crystals were flash-frozen in liquid N_2_.

Diffraction data were collected on an XRD1 station of the synchrotron ELETTRA [33], Trieste, Italy (wavelength 1.000 Å, temperature 100 K, detector Pilatus 2 M), and processed with XDS [34]. Phase information was obtained by molecular replacement using the EtLSC apo structure (PDB ID: 6FRW) [21] as the input model for MOLREP [35]. The obtained starting model was iteratively refined with *Coot* [36], REFMAC5 [37], and PHENIX [38]. The quality of the model was assessed using MOLPROBITY [39]. The final refinement statistics for the structure are reported in Table 1.

The electron-density map and polder map [40] were calculated using REFMAC and PHENIX, respectively. Crystallographic figures were created using PyMOL (The PyMOL Molecular Graphics System, Version 2.20 Schroedinger, LLC).

## 4. Conclusions

In this study, we presented the crystal structure of EtLSC in complex with LBS. LBS, produced by EtLSC by soaking the crystals in a solution containing 0.5 M sucrose, binds to an unusual pocket that has not been previously reported. The pocket is a plausible site of interaction for LBS and fructose-like intermediates and could therefore be relevant to FOS production in EtLSC. This pocket contains residues conserved across *Erwinia spp.* and it may have a similar role in these bacteria.

Further studies (e.g., mutagenesis) are required to understand the possible relevance of the loop formed by residues 368–378, and the role of Arg377 in the determination of the product spectrum of EtLSC and EaLSC. This pocket could be a target for engineered LSCs with tuned specificity and/or increased yield of F_n_ and/or low-DP FOSs.

## Figures and Tables

**Table 1:** Data collection and refinement statistics.

	6RV5 *Erwinia tasmaniensis* levansucrase
Wavelength (Å)	1.000
Temperature (K)	100
Resolution range (Å)	45.21–1.58 (1.64–1.58)
Space group	*P* 4_1_2_1_2
*a*, *b*, *c* (Å) and α, β, γ (°)	127.886, 127.886, 58.268; 90, 90, 90
Total reflections	552,442 (86,048)
Unique reflections	162,091 (25,949)
Multiplicity	3.4 (3.3)
Completeness (%)	99.47 (99.95)
Mean I/sigma(I)	20.50 (2.91)
Wilson B-factor (Å^2^)	18.23
R-merge	0.02668 (0.2867)
R-meas	0.03773 (0.4055)
R-pim	0.02668 (0.2867)
CC1/2	0.999 (0.888)
Reflections used in refinement	66,162 (6515)
Reflections used for R-free	3263 (343)
R-work	0.1456 (0.2640)
R-free	0.1929 (0.2902)
CC (work)	0.966 (0.827)
CC (free)	0.951 (0.716)
Ligands atoms	97
Solvent molecules	431
Protein residues	412
RMS (bonds) (Å)	0.017
RMS (angles) (°)	2.02
Ramachandran favored (%)	96.34
Ramachandran allowed (%)	3.41
Ramachandran outliers (%)	0.24
Rotamer outliers (%)	1.87
Clashscore	5.97
Average B-factor (Å^2^)	26.33
Macromolecules	23.87
Ligands	42.23
Solvent	41.99

Statistics for highest-resolution shell shown in parentheses.

## Abbreviations

LSC	Levansucrase
FOS	Fructooligosaccharide
LBS	Levanbiose
INU	Inulosucrase
GH68	Glycosyl hydrolase family 68
HMW	High-molecular weight
LMW	Low-molecular weight
DP	Degree of polymerization
